# Multiparametric MRI of early tumor response to immune checkpoint blockade in metastatic melanoma

**DOI:** 10.1136/jitc-2021-003125

**Published:** 2021-09-23

**Authors:** Doreen Lau, Mary A McLean, Andrew N Priest, Andrew B Gill, Francis Scott, Ilse Patterson, Bruno Carmo, Frank Riemer, Joshua D Kaggie, Amy Frary, Doreen Milne, Catherine Booth, Arthur Lewis, Michal Sulikowski, Lee Brown, Jean-Martin Lapointe, Luigi Aloj, Martin J Graves, Kevin M Brindle, Pippa G Corrie, Ferdia A Gallagher

**Affiliations:** 1 Department of Radiology, University of Cambridge, Cambridge, UK; 2 Cancer Research UK Cambridge Centre, Cambridge, UK; 3 Department of Radiology, Addenbrooke's Hospital, Cambridge, UK; 4 Department of Oncology, Cambridge University Hospitals NHS Foundation Trust, Addenbrooke's Hospital, Cambridge, UK; 5 Clinical Pharmacology & Safety Sciences, AstraZeneca PLC, Cambridge, Cambridgeshire, UK; 6 Department of Nuclear Medicine, Addenbrooke's Hospital, Cambridge, UK; 7 Cancer Research UK Cambridge Research Institute, Cambridge, UK

**Keywords:** biomarkers, tumor, CTLA-4 antigen, immunotherapy, melanoma, translational medical research

## Abstract

**Background:**

Immune checkpoint inhibitors are now standard of care treatment for many cancers. Treatment failure in metastatic melanoma is often due to tumor heterogeneity, which is not easily captured by conventional CT or tumor biopsy. The aim of this prospective study was to investigate early microstructural and functional changes within melanoma metastases following immune checkpoint blockade using multiparametric MRI.

**Methods:**

Fifteen treatment-naïve metastatic melanoma patients (total 27 measurable target lesions) were imaged at baseline and following 3 and 12 weeks of treatment on immune checkpoint inhibitors using: T_2_-weighted imaging, diffusion kurtosis imaging, and dynamic contrast-enhanced MRI. Treatment timepoint changes in tumor cellularity, vascularity, and heterogeneity within individual metastases were evaluated and correlated to the clinical outcome in each patient based on Response Evaluation Criteria in Solid Tumors V.1.1 at 1 year.

**Results:**

Differential tumor growth kinetics in response to immune checkpoint blockade were measured in individual metastases within the same patient, demonstrating significant intertumoral heterogeneity in some patients. Early detection of tumor cell death or cell loss measured by a significant increase in the apparent diffusivity (D_app_) (p<0.05) was observed in both responding and pseudoprogressive lesions after 3 weeks of treatment. Tumor heterogeneity, as measured by apparent diffusional kurtosis (K_app_), was consistently higher in the pseudoprogressive and true progressive lesions, compared with the responding lesions throughout the first 12 weeks of treatment. These preceded tumor regression and significant tumor vascularity changes (K^trans^, v_e_, and v_p_) detected after 12 weeks of immunotherapy (p<0.05).

**Conclusions:**

Multiparametric MRI demonstrated potential for early detection of successful response to immune checkpoint inhibitors in metastatic melanoma.

## Background

Immune checkpoint inhibitors targeting the cytotoxic T-lymphocyte antigen-4 (CTLA-4), programmed cell death receptor-1 (PD-1) and programmed cell death receptor-1 ligand (PD-L1) are improving outcomes for increasing numbers of patients with solid cancers.^1^ These drugs are now the standard of care for treating many cancers including metastatic melanoma.^2^ International trials testing anti-PD-1 antibodies alone or in combination with anti-CTLA-4 antibodies in metastatic melanoma reported objective response of up to 58% and only a complete response of 11.5% at a median follow-up of 12.2–12.5 months.^3^ Although durable remissions are achieved in some patients, approximately half of treated patients do not respond, while all treated patients are at risk of immune-mediated toxicity that can be both life changing and life threatening.^4 5^ In clinical practice, standard CT and MRI imaging are used for evaluation of treatment response, usually undertaken at 12 weekly intervals. Assessment of response in the first few months can be difficult and can be confounded by possible pseudoprogression, characterized by the enlargement of target measurable metastases followed by subsequent regression over time. Biomarkers that could aid clinical decisions in the early stages of treatment are currently lacking.^6^

Biomarkers derived from whole blood sampling and tumor biopsy do not reflect the spatiotemporal dynamics of tumor immune response to checkpoint inhibition due to the marked interpatient, intermetastatic and intratumoral heterogeneity present in melanoma.^7 8^ Pseudoprogression seen in a small number of patients receiving immune checkpoint inhibitors is difficult to distinguish from true tumor progression using size measurements alone on conventional CT.^9 10^ Functional imaging techniques have the potential to longitudinally characterize individual tumor response to immunotherapy and could potentially be used in the future to provide an early and accurate prediction of treatment response.

Several approaches have been investigated to date for imaging response to immune checkpoint inhibition. Positron emission tomography (PET) with the glucose analog 2-deoxy-2-[^18^F]fluoro-D-glucose (^18^F-FDG) has shown promise for long-term successful response monitoring: a complete metabolic response (CMR) with ^18^F-FDG uptake 1 year after commencing treatment is associated with an excellent progression-free survival compared with those patients who do not show CMR.^11^ However, it is not known whether ^18^F-FDG PET can detect early response to treatment, as it can be particularly difficult to distinguish tumor metabolism from glucose uptake associated with immune infiltration after the initial introduction of immune checkpoint inhibitors.^12^ Although zirconium-89 radiolabeled antibodies targeting CD8, PD-1, and PD-L1 have been developed as tracers for first-in-human trials in experimental medicine studies,^13–15^ these radiolabeled approaches are expensive and cannot be easily implemented as routine clinical tools.

MRI is a widely available clinical imaging tool. The technique is particularly well suited for longitudinal tracking of early treatment response, as it does not involve exposure to ionizing radiation.^16 17^ Dynamic contrast-enhanced MRI (DCE-MRI) measures properties of tissue vasculature^18^ and is increasingly used in the diagnosis, staging, and treatment response assessment of many cancers.^19^ Pharmacokinetic modeling of the T_1_-weighted contrast-enhanced images provide quantitative measurements of tissue perfusion and vascular permeability (see online supplemental material for a detailed explanation of these parameters). For example, K^trans^ is the volume transfer coefficient from the blood plasma space into the extravascular tumor interstitial space reflecting vascular permeability, which has been shown to change following successful treatment in a number of cancer types and therapeutic regimens.^20 21^ v_e_ is the fractional volume of the extravascular–extracellular space, and v_p_ is the vascular plasma volume. Following immunotherapy, DCE-MRI has been shown to detect tumor perfusion or vascular permeability as a surrogate biomarker of early tumor immune rejection in preclinical models of adoptive T cell therapy^22 23^ and has been foun to distinguish pseudoprogression from true tumor progression in patients with previously irradiated melanoma brain metastases after three cycles of ipilimumab.^24^

10.1136/jitc-2021-003125.supp1 Supplementary data

Diffusion-weighted imaging (DWI) is a complementary approach based on the molecular movement of water in tissues, which has been widely used for probing changes in cell density due to tumor cell death that occur following successful treatment in cancer.^17 25^ An advanced DWI approach termed diffusion kurtosis imaging (DKI) has been shown to detect tumor cellularity and heterogeneity in many cancer types based on the non-Gaussian movement of water within the heterogeneous tumor microenvironment.^26 27^ Cell density can be quantified on DKI based on the apparent diffusivity of water (D_app_), and the microscopic heterogeneity of this water diffusion in tissue can be probed using a dimensionless metric termed apparent diffusion kurtosis (K_app_). A more detailed explanation of these parameters can be found in online supplemental material.^27 28^

Here, we have used a multiparametric imaging approach combining morphological volumetric measurements with DCE and DKI to phenotype the microstructural and functional changes that occur in melanoma metastases before, during, and after treatment with immune checkpoint inhibitors.

In this prospective study, early changes in the growth kinetics, cellularity, heterogeneity and vascularity of the tumor microenvironment following immune checkpoint blockade between patients and between intermetastatic lesions were evaluated using multiparametric MRI (mpMRI). Metastatic melanoma offers a paradigm model to test the feasibility of these imaging methods in patients undergoing cancer immunotherapy.

## Methods

### Study design

Patients were recruited for mpMRI as part of the MelResist study, which evaluated response and resistance biomarkers in metastatic melanoma patients undergoing systemic therapy. Written informed consent was obtained from all patients before enrolment. Patient eligibility criteria for undertaking MRI included: (A) clinical diagnosis of unresectable and previously untreated metastatic melanoma (American Joint Committee on Cancer Stage IV); (B) a treatment plan to commence standard immune checkpoint inhibitors as first-line therapy for unresectable metastatic melanoma; (C) Eastern Cooperative Oncology Group performance status score of 0 or 1 and life expectancy of 12 weeks or greater; (D) measurable disease on baseline CT (tumor diameter >1 cm); (E) availability of recent excised or biopsied tissue samples from metastatic tumors for histopathological confirmation; (F) known BRAF V600 mutation status; and (G) no contraindication to undertaking MRI.

**Figure 1:**
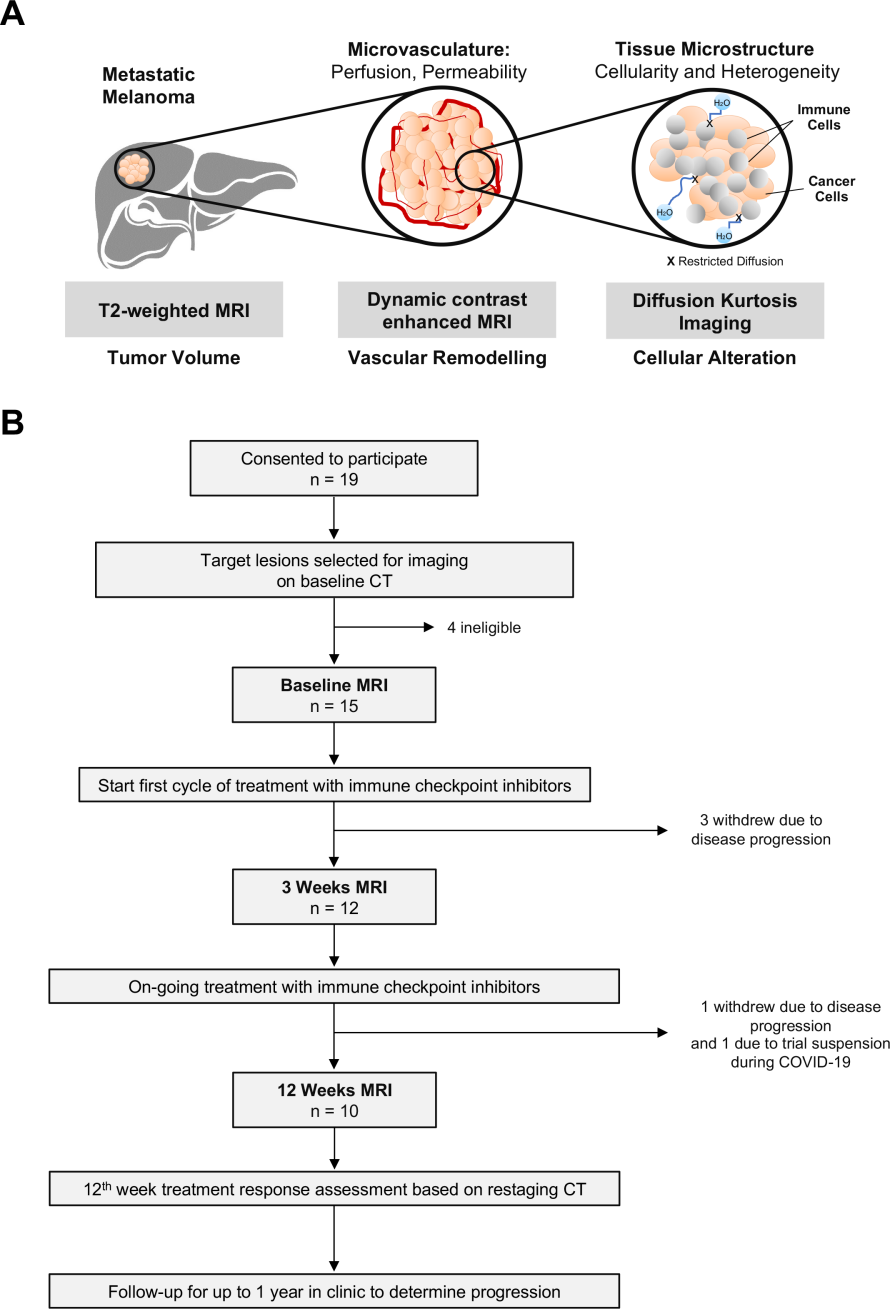
An mpMRI approach for longitudinal tracking of biological changes within tumors in response to immune checkpoint blockade. (A) Schematic diagram of the mpMRI approaches used in this study for monitoring tumor response to immune checkpoint blockade. K^trans^ measurements on dynamic contrast-enhanced MRI were used to quantify vascular permeability, while v_e_ and v_p_ reported on the volume of the extravascular–extracellular and vascular spaces, respectively. Diffusional kurtosis imaging, as an advanced form of diffusion-weighted imaging, was used to probe tissue microstructure using the metrics of apparent diffusivity (D_app_) as a measure of cellularity and apparent kurtosis (K_app_) for tissue heterogeneity. (B) Study flow chart for the melanoma immunotherapy trial (MelResist). mpMRI, multiparametric MRI.

Enrolled patients received one of the following regimens: (A) anti-PD-1 monotherapy, 2 mg/kg or 200 mg flat dose of pembrolizumab (Keytruda) every 3 weeks; or 3 mg/kg or 240 mg of nivolumab (Opdivo) every 2 weeks or 480 mg every 4 weeks and (B) combined anti-CTLA-4 and anti-PD-1 therapy, 3 mg/kg of ipilimumab (Yervoy) plus 1 mg/kg of nivolumab (Opdivo) every 3 weeks for four cycles followed by nivolumab 240 mg every 2 weeks or 480 mg every 4 weeks. All treatments were administered by intravenous infusion. Treatment continued until disease progression (as defined by the 3 monthly restaging CT scans), development of unacceptable adverse side effects such as autoimmune disorders or patient withdrawal of consent. A schematic diagram for the mpMRI study flow chart and the clinical characteristics of the study participants are as shown in figure 1 and table 1. Further details on the patient demographics can be found in online supplemental table S1.

**Table 1:** Clinical characteristics of study participants

Characteristics	
No. of patients	15
Age (median age, range)	65.4 (69, 48–76)
Gender	10 males, 5 females
AJCC Stage	IV
ECOG performance status	
0	9
1	6
BRAF status	
BRAF V600 mutant	3
BRAF Wild-type	12
Serum LDH (IU/mL) at baseline	
Normal (<250)	10
Elevated (>250)	5
Neutrophils-to-lymphocyte ratio at baseline	
Normal (<5)	12
High (>5)	3
Immunotherapy, n (%)	
Pembrolizumab	6 (40.0)
Nivolumab	2 (13.3)
Combined ipilimumab and nivolumab	7 (46.7)
RECIST 1.1 CT evaluation at 12th week, n (%)	
Partial response	5 (33.3)
Stable disease	4 (26.7)
Progressive disease	6 (40.0)
RECIST 1.1 CT evaluation at 1 year, n (%)	
Complete response	3 (20.0)
Partial response	4 (26.7)
Progressive disease	8 (53.3)
Anatomical site selected for MRI	
Head and neck	2
Chest	1
Abdomen and pelvis	7
Subcutaneous	4
Limbs	1

All patients imaged were histologically confirmed as AJCC Stage IV melanoma.

AJCC, American Joint Committee on Cancer staging (seventh edition); ECOG, Eastern Cooperative Oncology Group; LDH, lactate dehydrogenase; RECIST 1.1, Response Evaluation Criteria in Solid Tumors guidelines V.1.1.

### Magnetic resonance imaging

All patients underwent proton (^1^H) MRI on a 3.0 Tesla system (Discovery MR750, GE Healthcare, Waukesha, Wisconsin, USA) using a 32-channel phased-array coil with respiratory gating or multiple breath-holds used to reduce motion artifacts during image acquisition for lesions in the abdomen. The mpMRI protocol included multiplanar T_2_-weighted single-shot fast spin-echo anatomical imaging, DKI of tumor cellularity and heterogeneity, and DCE-MRI of tumor perfusion or vascular permeability. Imaging was conducted at three timepoints: within 1 week prior to starting treatment with immune checkpoint inhibitors (baseline MRI); 3 weeks after the first infusion (3-week MRI) and 12 weeks after the start of treatment (12-week MRI) coinciding approximately with the first standard-of-care restaging CT response assessment at 12 weeks. Further details on the imaging acquisition, image processing and analysis can be found in the online supplemental material and table S2.

### Classification of target melanoma metastases and measurement of response

Conventional objective response of the target metastases was determined by measuring the best treatment outcome at the 12-week restaging CT and reassessed at 1 year if the patient survived. Response was evaluated by standard Response Evaluation Criteria in Solid Tumors (RECIST) V.1.1 guidelines at 12 weeks and 1 year.^29^

In addition, tumor measurements were assessed using MRI during the first 12 weeks. Metastases with at least a 30% decrease in volume on the 12-week MRI were classified as responding, metastases with at least a 20% increase in volume were identified as true progression, while metastases with at least a 20% increase in volume at the 3-week MRI, but which subsequently decreased in >30% on the 12-week scan, were classified as pseudoprogression.

### Statistical analysis

Statistical analysis was performed in GraphPad Prism software V.8 (La Jolla, California, USA). All values were expressed as median and IQR to account for sample size differences between groups. Normality was assessed using the Shapiro-Wilk test. Changes in individual lesion mpMRI biomarkers over the treatment timepoints were evaluated using either paired t-test for normally distributed data or Wilcoxon matched-pair signed-rank test for data with non-parametric distribution. Differences between the subgroups of responding, pseudoprogressive, and true progressive lesions were evaluated using one-way analysis of variance for normally distributed data, or the Kruskal-Wallis test with Dunn’s multiple comparison for non-parametric testing. Spearman’s correlation analysis was used for evaluating any relationship between the mpMRI biomarkers across treatment timepoints. A value of p<0.05 was considered as statistically significant.

## Results

### Clinical characteristics

Fifteen treatment-naïve patients (10 males, 5 females; median age 65 years) were imaged with mpMRI over the first 12 weeks of immunotherapy. Ten patients completed MRI at all three imaging timepoints (baseline, 3-week and 12-week MRIs); 5 patients were scanned at baseline and/or 3 weeks before withdrawal from the trial due to clinical reasons such as early disease progression or clinical deterioration incompatible with continuing on the study. An additional four patients were enrolled on the study but were deemed as ineligible for the prospective trial due to insufficient time for scheduling of imaging scans before the start of treatment (within a week) or target lesions that were too small (less than 1 cm in largest diameter) for multiple timepoint imaging and follow-up treatment response assessment. 53% of the patients received PD-1 monotherapy, while 47% of the patients were treated with combined CTLA-4 and PD-1 therapy.

### Differential response to immune checkpoint blockade

Based on RECIST V.1.1 assessment at the 12-week restaging CT, five patients demonstrated partial response to immune checkpoint inhibitors, four had stable disease, and six showed disease progression. The patients with stable disease demonstrated differential response between the individual metastases. Two out of these four patients subsequently progressed at the 1-year restaging CT, and the remaining two patients demonstrated continued response to treatment (table 1). Consequently, at the 1-year timepoint, three patients showed complete response, four demonstrated partial response, one was alive with progressive disease, and the remaining seven had died from progressive disease. Further details on patient demographics can be found in online supplemental figure 1.

The mpMRI images for a total number of 27 enhancing target melanoma metastases that were first identified as more than 1 cm in diameter on staging CT were analyzed. In this study, a total of 13 responding, 4 pseudoprogressive, and 10 true progressive metastases were identified by MRI. Tumors were categorized into three subgroups (responding, pseudoprogression, and true progression) were based on comparing the 3-week MRI with the 12-week MRI, confirmed with restaging CT at 12 weeks and follow-up on the clinical outcome for up to 1 year. There were no lesions that showed a 30% decrease in volume at the 3-week MRI, which subsequently increased in volume at the 12-week MRI or on the restaging CT.

**Figure 2:**
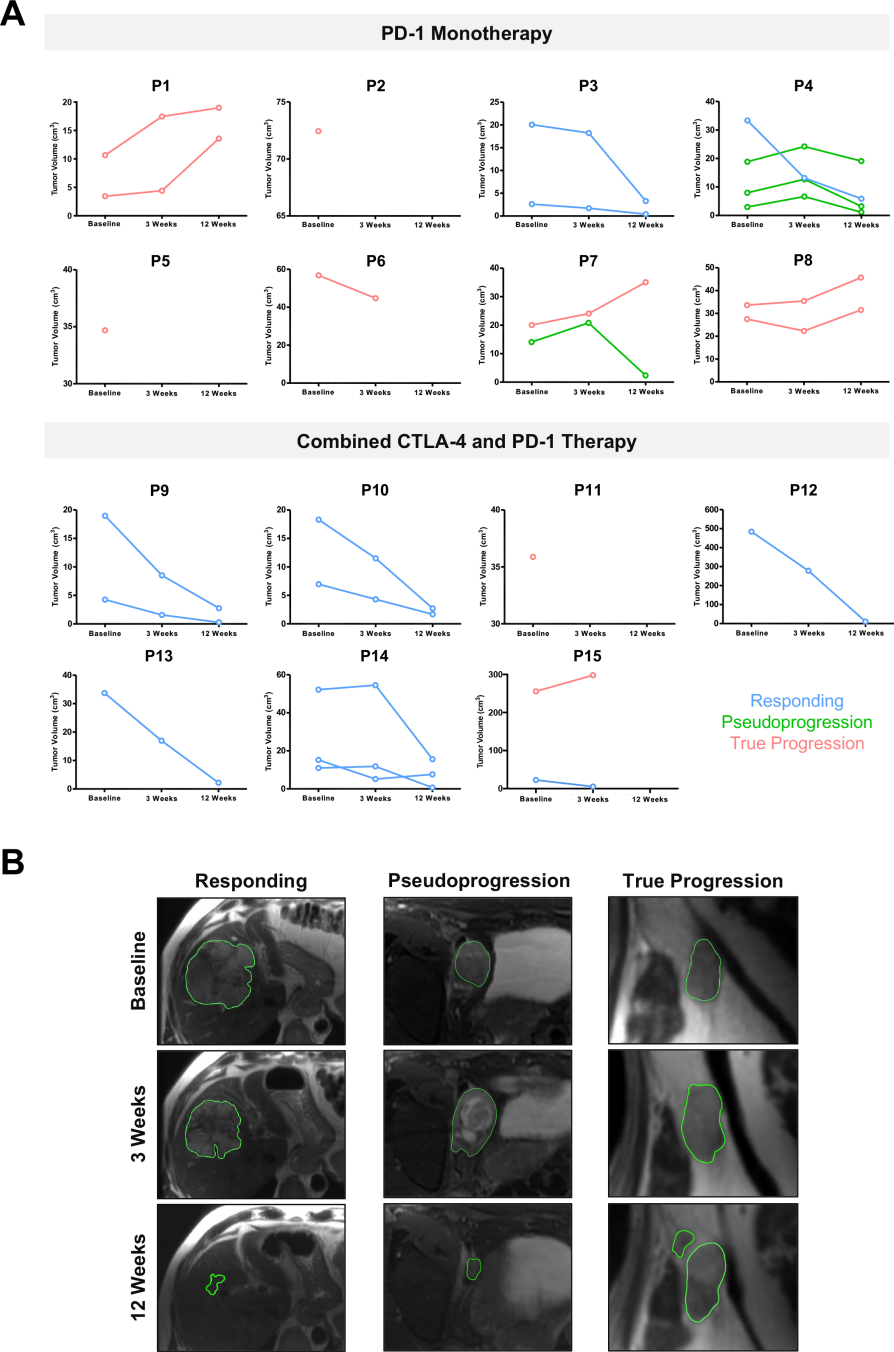
Interpatient and intermetastatic heterogeneity in response to immune checkpoint blockade. (A) Differential tumor growth kinetics in patients receiving PD-1 monotherapy compared with combined CTLA-4 and PD-1 treatment. Individual tumor volumes were measured on T_2_-weighted MRI. Categorization of tumors into three subgroups (responding, pseudoprogression, and true progression) were based on comparing the 3-week MRI with the 12-week MRI, confirmed with restaging CT at 12 weeks and follow-up on the clinical outcome for up to 1 year. (B) Representative T_2_-weighted images from three patients with the classic features of responding, pseudoprogressive, and true progressive lesions. Note the T2 hyperintensity in keeping with inflammation in the pseudoprogressive lesion at 3 weeks. CTLA-4, cytotoxic T-lymphocyte antigen-4; PD-1, programmed cell death receptor-1.

**Figure 3:**
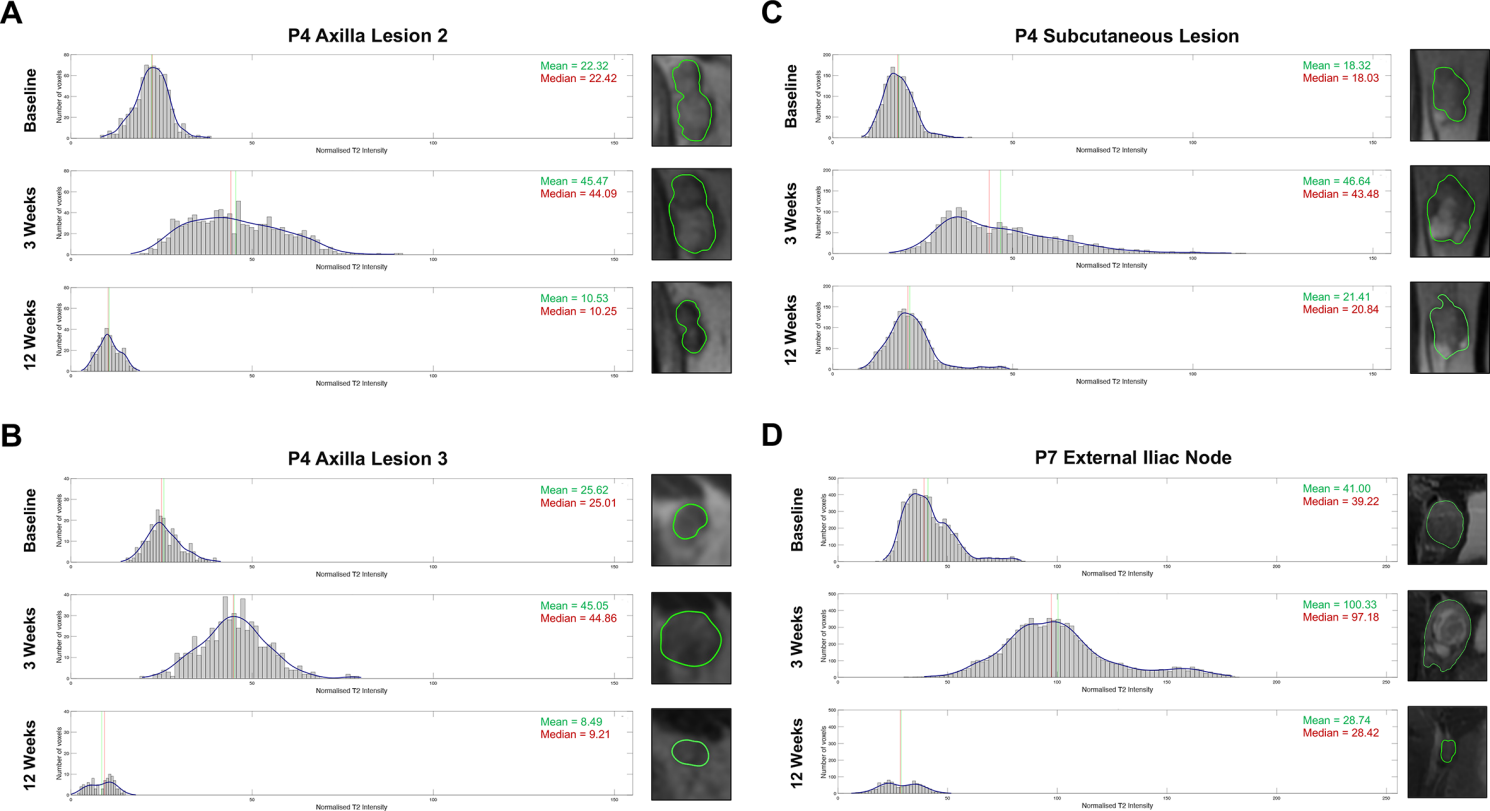
Histogram analysis of the T_2_ intensity values of all four pseudoprogressive lesions.^8^ These included: the (A) axilla lesion 2, (B) axilla lesion 3, (C) subcutaneous lesion of patient 4, and the (D) external iliac node of patient 7.

T_2_-MRI volumetric analysis showed differential interpatient and intermetastatic response to immune checkpoint blockade. Within the cohort of patients in our study, intermetastatic differences in the individual tumor growth kinetics were particularly evident in patients undergoing anti-PD-1 monotherapy, as compared with patients receiving combined CTLA-4 and PD-1 treatment, where response was almost immediate at the 3-week MRI (figure 2A), which may represent the fact that monotherapy takes longer to mount antitumor effects compared with combination therapy. Interestingly, increasing T_2_ hyperintensity or inflammatory changes were detected within all four enlarged pseudoprogressive tumors at 3 weeks, which resolved at 12 weeks with a corresponding reduction in tumor volume (figure 2B and figure 3).

### Tumor cell death and changes in heterogeneity in response to treatment

**Figure 4:**
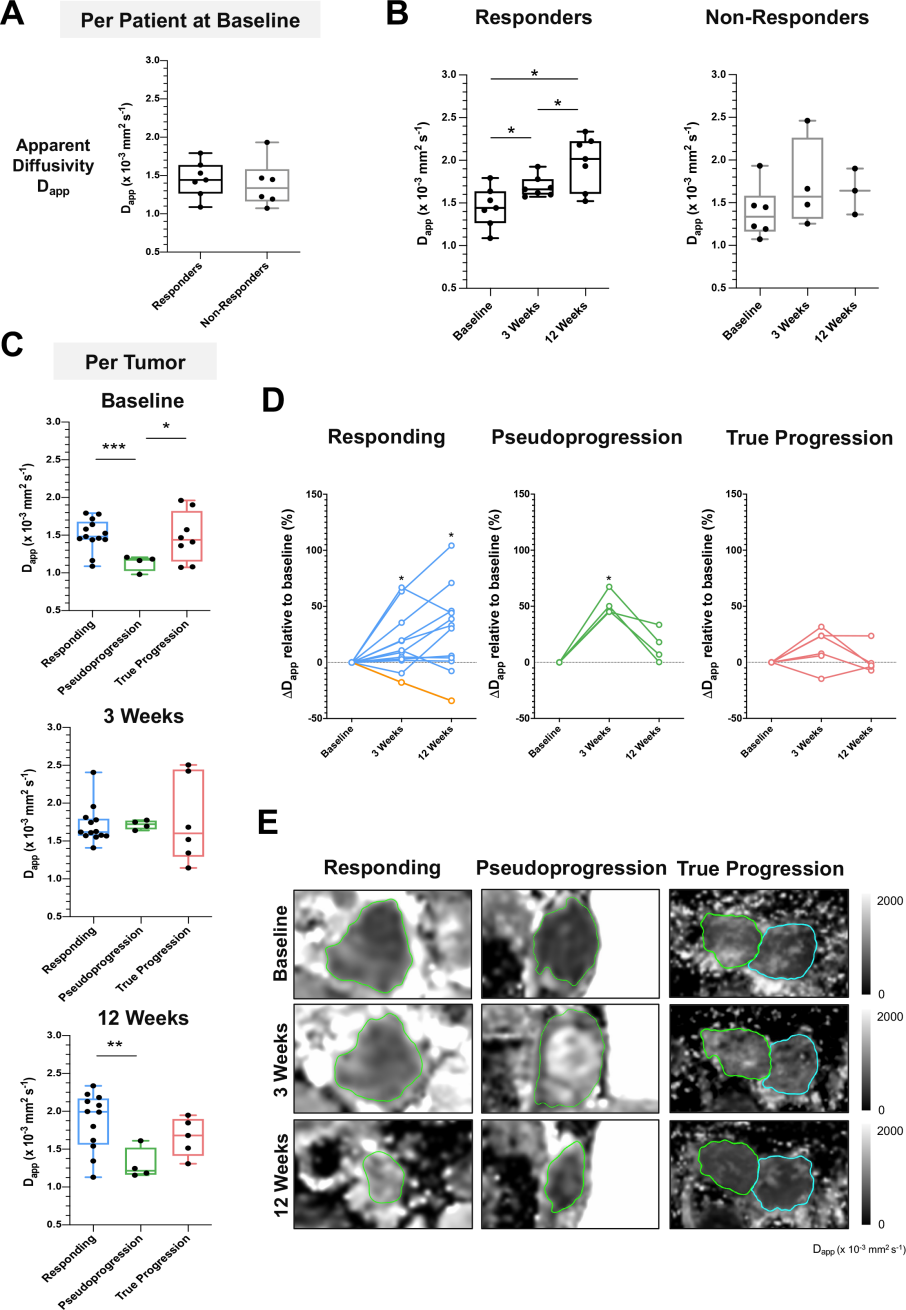
Early detection of tumor cell death using DKI. (A) Comparison of apparent diffusivity (D_app_) as a measure of tumor cell density between responders and non-responders at baseline before the start of treatment. (B) Changes in average tumor D_app_ on a per patient basis over the course of treatment, divided according to overall response. (C) Response of individual lesions classified into three subgroups (responding, pseudoprogression, and true progression) showing the differences in tumor cellularity at baseline. (D) Percentage change in D_app_ relative to baseline in individual lesions from the three subgroups. (E) Representative D_app_ images from three lesions categorized as responding, pseudoprogression, and true progression, respectively, based on the 1-year restaging CT. Data are presented as median and IQR. Normality was assessed using the Shapiro-Wilk test. Mann-Whitney test was performed to assess differences between two independent lesion subgroups; Kruskal-Wallis test with post hoc Dunn’s multiple comparison analysis was performed to test for differences between three independent lesion subgroups; *p<0.05; ***p<0.001. Yellow line in figure part D indicates the percentage change in D_app_ for patient 4. Analysis of apparent kurtosis as a measure of tumor heterogeneity, detected concurrently using DKI, is found in online supplemental figure S1. DKI, diffusion kurtosis imaging.

Figure 4 and online supplemental figure S1 show the changes in tumor cellularity and heterogeneity measured on DKI. No significant difference in the average D_app_ for each patient, as a measure of tumor cell density, was detected between the responding and non-responding patients at baseline (figure 4A); median D_app_ of 1.44 for responding patients versus 1.33 for non-responding patients, p=0.62). There was a significant increase in the average D_app_ of imaged target metastases for each patient representing reduced tumor cellularity (p<0.05) in the responding patients at 3 weeks (median D_app_ 1.65; IQR 1.59–1.77) compared with baseline (1.44; IQR 1.26–1.63), with a further significant increase at 12 weeks (2.01; IQR 1.60–2.22). In contrast, there was no significant change in D_app_ in the tumors of non-responders over the 12 weeks of treatment (figure 4B).

**Figure 5:**
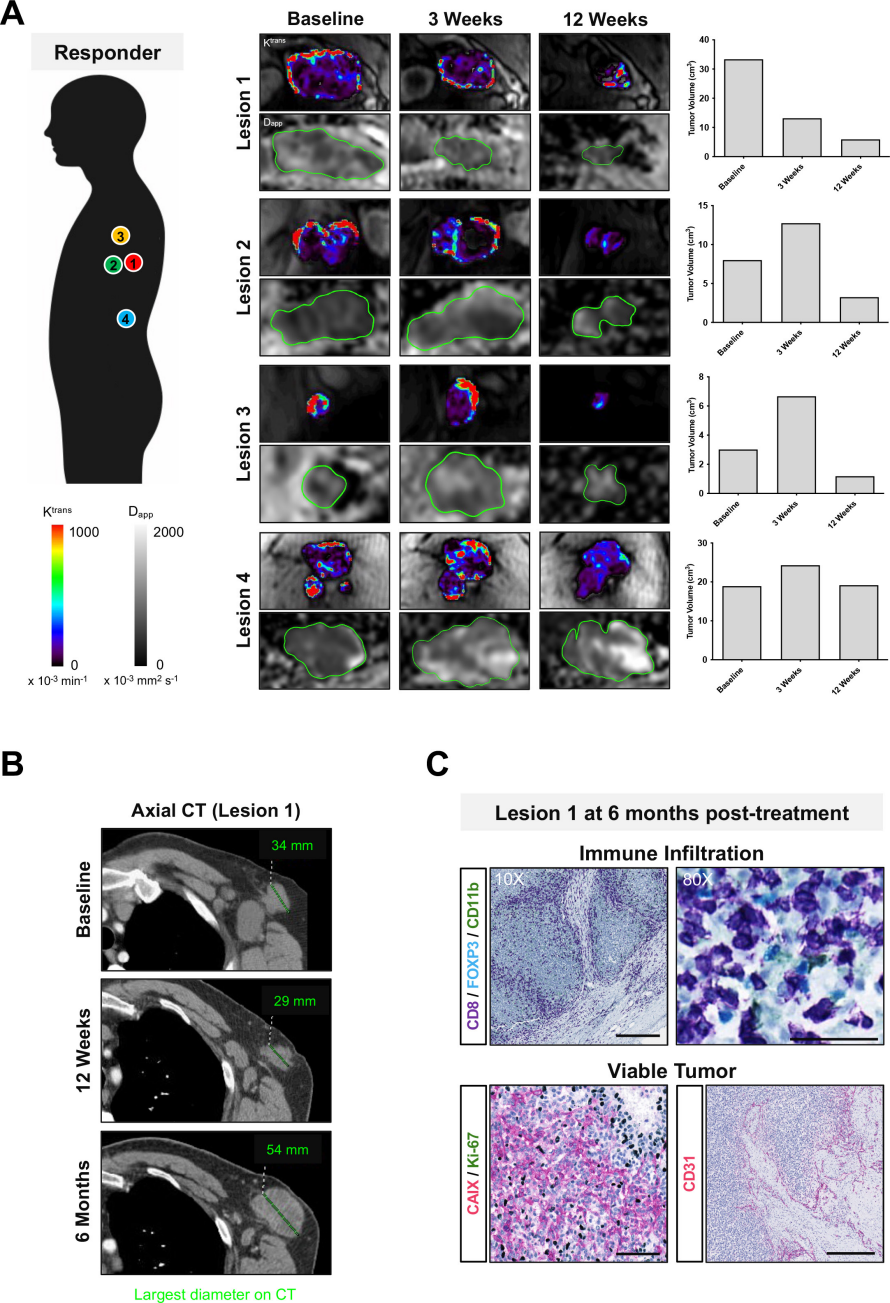
Dynamic changes in intertumoral response to immune checkpoint blockade within a single patient. (A) Multiple metastases in a patient on treatment with nivolumab (PD-1 monotherapy). Intertumoral differences in treatment response, vascular permeability, and cellularity were measured in four target lesions on MRI during the first 12 weeks of treatment. Increased cellularity was detected in the responding tumor (lesion 1) despite a reduction in tumor volume and lower vascularity at 12 weeks. The responding lesion subsequently progressed at 6 months on treatment and was surgically resected. (B) Axial CT of lesion 1 at baseline, 12 weeks, and 6 months; largest tumor diameter shown in mm. (C) Immunohistochemistry of lesion 1 showed remarkable infiltration of immune cells (CD8) in viable tumor tissues that were highly hypoxic (CAIX), proliferative (Ki67), and vascular (CD31). Scale bars for CD8 immunostained images represent 100 µm (10× magnification) and 50 µm (80× magnification); 100 µm in CAIX/Ki67 dual staining (20× magnification) and CD31 (10× magnification). PD-1, programmed cell death receptor-1.

Further analysis based on classification of individual metastases from all patients into the three subgroups of ‘responding’, ‘pseudoprogression’ and ‘true progression’, showed a significantly lower D_app_, reflecting higher tumor cell density at baseline in the pseudoprogressive lesions (median 1.17; IQR 1.02–1.20), as compared with the responding (median 1.48; IQR 1.44–1.68; p<0.001) and true progressive lesions (median 1.44; IQR 1.15–1.82; p<0.05). Individual tumors responded differently to treatment: most of the responding and pseudoprogressive lesions exhibited a significant percentage increase in D_app_ at the 3-week MRI relative to baseline, indicating lower cellularity in most responding lesions (median increase in D_app_ by 8.9%; IQR 2.3%–27.6%; p<0.05) and pseudoprogressive lesions (median increase by 48.0%; IQR 45.2%–63.1%; p<0.05). A further increase in D_app_ was detected within the tumor microenvironment in most of the metastases responding at 12 weeks (31.7%; IQR 1.9%–45.5%; p<0.05). However, one lesion demonstrated higher cellularity (increase in D_app_) despite a reduction of tumor volume over 12 weeks of treatment; interestingly, this lesion subsequently increased in size at the sixth month restaging CT and was verified to be a pseudoprogressive lesion over a longer timeframe. Higher cellularity was also detected on average in the pseudoprogressive lesions at 12 weeks compared with the responding lesions, despite a reduction in tumor volume, which may reflect a later phase of immune infiltration and tumor cell killing in these metastases (figures 4 and 5). This patient demonstrated complete response on RECIST V.1.1 evaluation at the 1-year follow-up CT scan. Overall, the D_app_ values measured from the experimental DKI images correlated to the apparent diffusion coefficient (ADC) values obtained on clinical DWI for all imaged patients (online supplemental figure S2).

No significant difference in microscopic tumor heterogeneity at baseline (p=0.27) was detected in the tumors of the responders (median K_app_ 0.61; IQR 0.51–0.71), compared with non-responding patients (median 0.71; IQR 0.53–0.86), as detected by the average apparent kurtosis (K_app_) values from all target lesions for each patient obtained concurrently on DKI (online supplemental figure S1). A significant reduction in K_app_ (p<0.05) was detected in the tumors of responding patients at 3 weeks (median 0.59; IQR 0.51–0.65) compared with baseline (median 0.61; IQR 0.51–0.71). Further analysis of the individual lesions showed a trend towards a higher level of tumor heterogeneity, as measured by K_app_, in the pseudoprogressive lesions throughout the first 12 weeks of treatment (baseline: 0.84; 3 weeks: 0.64; 12 weeks: 0.76) compared with the responding lesions (baseline: 0.59; 3 weeks: 0.54; 12 weeks: 0.56); although this did not reach statistical significance, it may reflect underlying immune cell infiltration or cell death over the course of treatment. As with the results for D_app_, no significant change in K_app_ was detected in the progressing metastases during the first 12 weeks of treatment.

### Tumor vascular remodeling following cell death

**Figure 6:**
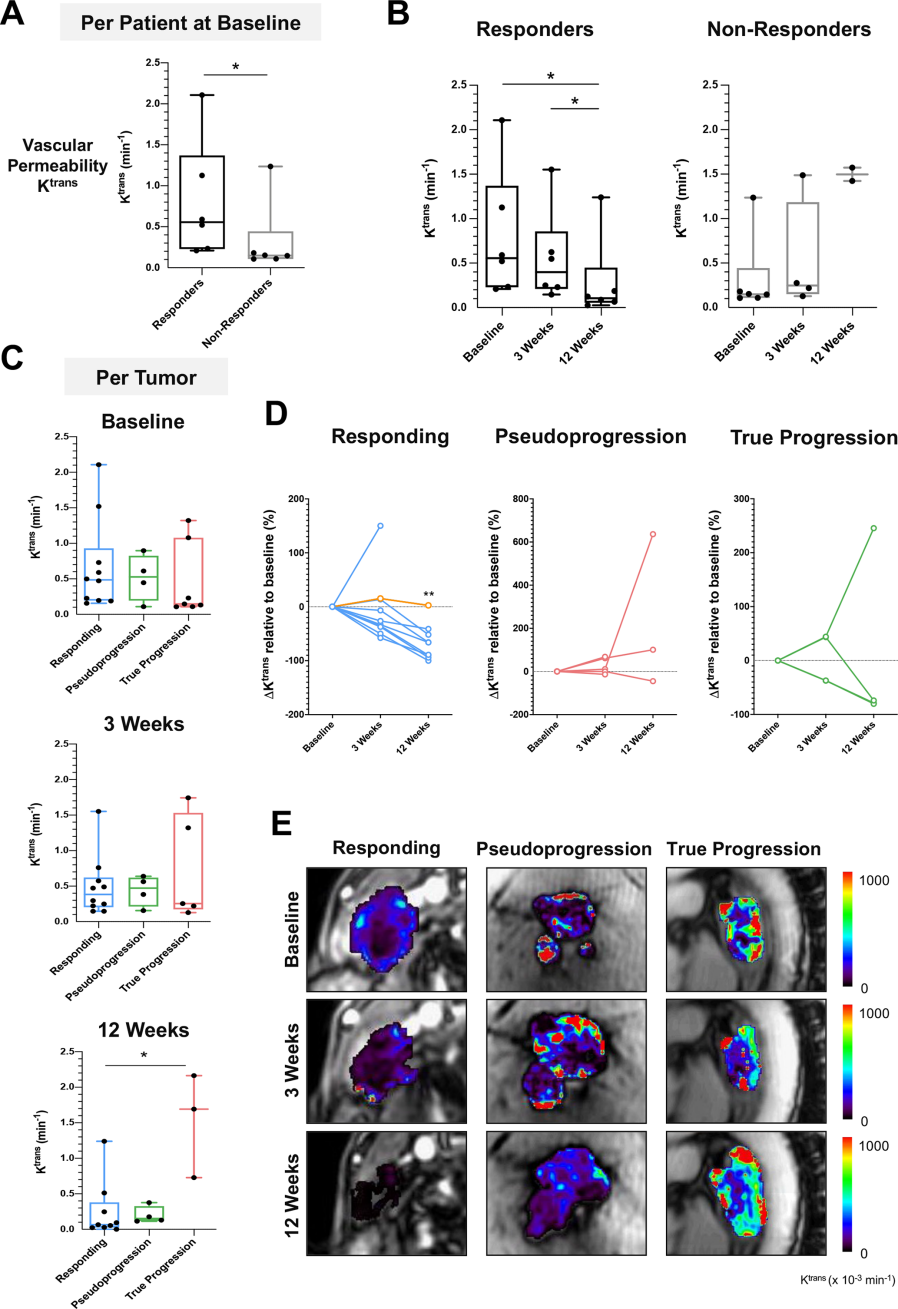
Tumor vasculature remodeling following immune cytotoxic killing and tumor cell death. (A) Comparison of vascular permeability K^trans^ between responders and non-responders at baseline before the start of treatment. (B) Changes in tumor K^trans^ over the course of treatment. (C) Comparison of tumor vascular permeability at baseline between individual lesions from the three subgroups: responding, pseudoprogression, and true progression. (D) Percentage change in K^trans^ relative to baseline in individual lesions from the three subgroups. (E) Representative K^trans^ images from the three subgroups lesions. *P<0.05; **p<0.01. Yellow line in figure part D indicates the percentage change in K^trans^ for patient 4. Analysis for other DCE-MRI parametric measurements is found in online supplemental figure S3. DCE-MRI, dynamic contrast-enhanced MRI.

Figure 6 and online supplemental figure S3 show the changes in tumor vascularity and perfusion during 12 weeks of treatment, as measured by DCE-MRI and contrast kinetic modeling using the extended Tofts model. The average tumor vascular transfer constant (K^trans^) at baseline was higher in the target lesions of the responding patients (median K^trans^ 0.56; IQR 0.23–1.37) compared with the non-responders (0.15; IQR 0.11–0.44; p<0.05). Similarly, the average fractional extravascular–extracellular volume (v_e_) at baseline of the target lesions of the responding patients were higher (median v_e_ 0.49; IQR 0.31–0.77) compared with the non-responders (0.19; IQR 0.15–0.32; p<0.05). A significant reduction in these tumor vascularity metrics (K^trans^, v_e_, and v_p_) was only detected at 12 weeks compared with baseline (0.11; IQR 0.05–0.45; p<0.05) but not at 3 weeks. A gradual increase in tumor vascular metrics was detected in the tumors of non-responding patients over the course of treatment, but this was not statistically significant, which may reflect the small numbers, particularly at the 12-week MRI. Further analysis on the individual lesions showed no significant difference in the vascular transfer constant K^trans^, fractional volume of the extravascular–extracellular space v_e_, or fractional plasma volume v_p_, between the three subgroups of lesions before the start of treatment. A significant decrease in K^trans^ relative to baseline was detected in most responding lesions at 12 weeks (median −66.19%; IQR −92.00 to −46.49%; p<0.01) but not at the 3-week MRI (−29.73%; IQR −40.51 to 14.01%; p=0.23). Similarly, v_e_ and v_p_ were also lower in the responding lesions at 12 weeks (p=0.07 and p<0.01, respectively). A trend towards lower K^trans^ was also detected in most pseudoprogressive lesions at 3 weeks (median 0.47; IQR 0.18–0.60) and 12 weeks (0.15; IQR 0.11–0.32), compared with baseline (0.52; IQR 0.19–0.82), but this was not statistically significant given the small number of pseudoprogressive lesions within the patient cohort.

### Early treatment timepoint changes in tumor cellularity is independent of tumor volume

Spearman’s correlation analysis of the mpMRI biomarkers over the first 12 weeks of treatment in this cohort showed no significant correlation between tumor volume and D_app_ (online supplemental figure S4). This implies that the early detection of changes in tumor cellularity from immune cytotoxic killing of tumor cells at the 3-week MRI was independent of changes in tumor volume. However, a positive correlation was found between tumor volume and all metrics of tumor vascularity and perfusion at 12 weeks (K^trans^, v_e_, and v_p_), suggesting that vascular remodeling may be related to tumor size changes following immune checkpoint blockade.

## Discussion

As immune checkpoint inhibitors become more widely used in routine clinical practice, there is an unmet need for more effective tools to measure successful response to these agents. This is increasingly important as more patients with cancer are being offered long term immunotherapy, which is costly to healthcare systems and comes with a significant risk of side effects. Tumor heterogeneity is one of the major challenges for effective cancer treatment and manifest as morphological, functional, cellular, metabolic, and molecular diversity.^30 31^ Clinical tools to image this multilayered tumor heterogeneity and how it changes with immunotherapy could have a role in differentiating tumor resistance from successful response early in the treatment pathway.^32^

In this study, longitudinal tracking of microstructural and functional changes in metastatic melanoma during the first 12 weeks of treatment was performed using mpMRI. Heterogeneity in response to immune checkpoint blockade was observed in these treatment-naïve tumors. This heterogeneity may be due to interpatient and intermetastatic differences in tumor immunogenicity, as some patients may have more delayed response, multiple waves of immune activation or ongoing immune evasion and clonal expansion during continuous treatment, as exemplified by patient 4. These changes in the tumor microenvironment may not be identified by standard CT or MRI, as the change in tumor size alone are often insufficient to determine treatment benefit at the early stages of immunotherapy. Therefore, a clinically applicable tool to evaluate immunotherapy is required to guide clinical decision making.^33^

Treatment response to immune checkpoint blockade was captured longitudinally on mpMRI in this study using three approaches to assess the tumor microenvironment: T_2_-weighted MRI of tissue structure, DKI of cellular density and its microscopic heterogeneity, and DCE-MRI of the tumor vasculature. An interesting and unexpected observation was an increase in median T_2_-weighted signal intensity following 3 weeks of immunotherapy (one infusion of immunotherapy) in the pseudoprogressive metastases, compared with metastases that responded or were shown to progress at later timepoints. This is likely to be due to tumor enlargement from significant immune cell infiltration and inflammation, rather than tumor proliferation. T_2_-weighted MRI represents a very simple routine clinical tool that may be able to discriminate pseudoprogression from true progression if this initial observation could be confirmed in larger studies. Quantitative analysis of these signal intensity changes, for example, using T_2_ mapping, may be useful in future trials for more detailed characterization of these microstructural changes.^34^

Changes in cell density were measured using D_app_ on DKI to detect either cytotoxic T-cell killing or increased cell density from immune infiltration or tumor proliferation. Cell loss measured as an increase in the median D_app_ was detected in both responding and pseudoprogressive lesions as early as 3 weeks after the start of treatment compared with baseline. Further reductions in cell density within the responding lesions were detected at the 12-week MRI. An increase in ADC measured on DWI (equivalent to the D_app_ measured here) has also been reported in previously treated ocular melanoma responding to immunostimulatory adenoviral CD40L gene therapy: ≥1 fold change in ADC at week 5 following treatment was a better predictor of objective survival than metabolic changes on ^18^F-FDG PET and tumor size changes on MRI.^35^ Interestingly, in our study, an increase in cell density (or lower D_app_) was detected in the pseudoprogressive lesions at 12 weeks compared with the responding lesions, despite reduced tumor volumes measured on the 12-week MRI and standard restaging CT. No significant correlation between D_app_ and tumor volume was detected across the imaging timepoints, which implies that the estimation of cell density based on water diffusion within the tumor microenvironment is independent of tumor volume and is therefore an important additional metric to measure. A higher degree of tumor heterogeneity, as assessed by an increase in K_app_, was detected in the pseudoprogressive lesions throughout the MRI imaging timepoints, compared with the responding lesions. This supports the hypothesis that there is underlying cellular alteration with different phases of immune activation and proliferation in the pseudoprogressive lesions over the course of treatment. Although there was a higher K_app_ in the true progressive lesions at all imaging timepoints compared with the responding lesions, the feasibility of using DKI alone to differentiate true progression from pseudoprogression could not be established as the number of non-responders with complete MRI scans are limited in the metastatic disease setting due to early disease progression and withdrawal from the trial. Nevertheless, greater tumor heterogeneity at baseline (entropy, dissimilarity, and contrast texture features) measured on CT radiomics has been previously reported in non-responders to PD-1 monotherapy.^36^

The tumor vasculature plays a significant role in regulating tumor homeostasis, metastasis, and immune trafficking.^37^ The vascular networks in malignant tumors are typically disorganized with immature, tortuous, and leaky blood vessels that are hyperpermeable to intravascular contrast agents. DCE-MRI showed a gradual decrease in: the tumor vascular permeability K^trans^; the extravascular–extracellular space v_e_; and the plasma volume fraction v_p_ within the responding lesions. This was more prominent at the 12-week MRI when a reduction in tumor burden was detected, suggesting that tumor vasculature remodeling and shutdown may have occurred following cell death caused by cytotoxic T cell killing. This contrasts with the effects of antiangiogenic treatments in human melanoma xenografts whereby the treatments are more directed towards the vascular network and are generally not cytotoxic. Tumor vasculature remodeling as represented by a lower K^trans^, often precedes cell death and reduction in tumor burden, with no significant change in cell density measurements such as v_e_ or ADC.^38^ Despite the small number of pseudoprogressive lesions available for analysis in our study, lower K^trans^, v_e_ and v_p_, with a corresponding reduction in tumor volume because of cell death, was detectable in most pseudoprogressive lesions at 12 weeks. The pseudoprogressive lesions in general demonstrated lower vascular permeability and perfusion compared with the true progressive lesions at 12 weeks. Our findings are in concordance with a previous study assessing DCE-MRI melanoma brain metastases study in which lower v_p_ was detected in previously irradiated pseudoprogressive lesions compared with true progressive lesions after three cycles of ipilimumab.^24^ This suggested that DCE-MRI may have utility in distinguishing true progressive lesions from treatment-responsive lesions but at a later timepoint compared with diffusion measurements. Interestingly, higher K^trans^ and v_e_ were detected at baseline in all imaged tumors of most patients who were responders to immune checkpoint inhibitors compared with the non-responders. One explanation could be that the differences in tumor vasculature between tumors may play a role in determining immune trafficking and subsequent immune eradication of tumor cells.^37^ Ideally, this could be explored by tissue sampling of multiple lesions both before and during therapy, but this is not practical in the metastatic disease setting clinically, and further preclinical research is required.

As part of this prospective study, we have demonstrated marked intralesional, intermetastatic, and interpatient heterogeneity in melanoma over the first 12 weeks of immunotherapy. After only 3 weeks of treatment or one infusion of immunotherapy, a decrease in cellularity, as measured on DKI, could distinguish responding patients from non-responders, as well as individual responding and pseudoprogressing tumors from true progressing ones. An interesting finding was an increase in normalized T_2_-weighted signal and its distribution in the pseudoprogressing lesions compared with the progressing lesions after 3 weeks treatment. Therefore, combining conventional T_2_-weighted and DKI at 3 weeks after starting immunotherapy could be used to identify pseudoprogression during the early stages of treatment. Although there was higher tumor vascular permeability and perfusion at baseline in the responding patients on DCE-MRI compared with non-responders, changes in K^trans^ could not be used to distinguish responding and pseudoprogressing lesions until after 12 weeks of treatment showing that measurable vascular changes occur later than changes in cellularity.

Our study presented several strengths and limitations. This is the first prospective MRI study to serially track cellular and functional changes in melanoma metastases during immune checkpoint blockade. As all melanoma metastases analyzed in this study were previously untreated and unresectable tumors, the treatment timepoint changes measured on mpMRI were directly related to immunotherapeutic effects on individual lesions. Partial volume effects on image measurements were minimal as several patients in our trial presented large metastases at baseline. A stringent criterion for imaging and analysis was maintained to include only patients with measurable disease so that the biological changes were trackable over 12 weeks of immunotherapy. Nevertheless, our study is limited by the small sample size, which restricts the scope for wider interpretation of the results and evaluation of the imaging biomarkers for their predictive values. Future multicenter trials are required to test and validate these imaging biomarkers in a larger patient cohort, with the aim of integrating these imaging methods into immunotherapy trials and routine clinical management. Our study is further limited by the lack of radiological–pathological correlation, as relatively few metastases are readily accessible to biopsy. This difficulty in obtaining tumor tissues from metastatic sites further highlights the strengths of non-invasive imaging as a surrogate for pathology as changes in tumor growth kinetics, cell density, heterogeneity, and vascularity within individual tumors could be longitudinally tracked over the course of treatment.

Although no CT was available at the 3-week MRI timepoint for direct comparison, the lesional volume on MRI at this early timepoint represents a surrogate for the CT size measurements. Using size criteria alone, conventional CT is unlikely to provide additional information over MRI. The latter not only provides enhanced soft tissue contrast, but importantly can probe quantitative measures of tissue function that are not possible with CT, thus providing significant biological information as we have demonstrated here. Although CT can be used to probe tumor perfusion as part of dynamic contrast-enhanced CT, this is usually at lower temporal resolution and with a significant radiation burden given the multiple acquisition timepoints. More generally, the radiation dose from CT decreases its suitability for multiple timepoint imaging, especially in patients who are particularly at risk of radiation effects. The emerging field of radiomics to study tissue heterogeneity would also be interesting to evaluate on CT at this early 3-week timepoint, but both radiomics and CT perfusion are currently research tools and require validation within future prospective trials.

In conclusion, mpMRI has shown potential for early assessment of response to immunotherapy in metastatic melanoma patients. Early changes in tumor cellularity measured on DKI following 3 weeks after starting treatment could be used to detect responding and pseudoprogressive melanoma metastases before a change in tumor volume and vascular permeability. This work could have important implications for monitoring treatment of metastatic melanoma and the increasing number of solid cancers treated with immunotherapy.

## Data Availability

Data are available on reasonable request. Data may be obtained from a third party and are not publicly available. All data relevant to the study are included in the article or uploaded as supplementary information. Please contact the corresponding author Doreen Lau (la399@cam.ac.uk).
