# Supplementary material for: Multiparametric MRI of early tumor response to immune checkpoint blockade in metastatic melanoma
Source: J Immunother Cancer. 2021 Sep 23;9(9):e003125. doi: 10.1136/jitc-2021-003125 (PMC8475139; doi:10.1136/jitc-2021-003125)
Supplement: Supplementary data [file jitc-2021-003125supp001.pdf]

Supplemental Material

Table S1. Patient demographics.

Patient	ECOG	BRAF Status	Treatment	RECIST 1.1 Evaluation of Standard Clinical CT		Body Site(s) Imaged	No. of Target Lesions Imaged	Research MRI Scan Completion		
				12 Weeks	1 Year			Baseline	3-Weeks	12-Weeks
P1	1	WT	Pembrolizumab	PD	* PD	Subcutaneous	2	Yes	Yes	Yes
P2	1	WT	Pembrolizumab	SD	* PD	Liver	1	Yes	Withdrew from study (claustrophobia and clinical deterioration)	
P3	0	V600 Mutant	Pembrolizumab	PR	PR	Inguinal and external iliac nodes	2	Yes	Yes	Yes
P4	1	WT	Nivolumab	SD	CR	Subcutaneous	4	Yes	Yes	Yes
P5	1	WT	Nivolumab	PD	* PD	Right occipital and posterior cervical region in the neck	1	Yes	Withdrew from study (disease progression)	
P6	1	WT	Pembrolizumab	PD	* PD	Lower limb	1	Yes	Yes	Withdrew from study (disease progression)
P7	0	WT	Pembrolizumab	SD	* PD	Inguinal and external iliac nodes	2	Yes	Yes	Yes

P8	0	WT	Pembrolizumab	PD	PD	Subcarinal/Paraesophageal nodes	2	Yes	Yes	Yes
P9	0	WT	Combined Ipilimumab and Nivolumab	PR	CR	Cervical lymph node and supraclavicular	2	Yes	Yes	Yes
P10	0	WT	Combined Ipilimumab and Nivolumab	PR	CR	Subcutaneous	2	Yes	Yes	Yes
P11	0	V600 Mutant	Combined Ipilimumab and Nivolumab	PD	* PD	Medial clavicle	1	Yes	Withdrew from study (operation and disease progression)	
P12	0	WT	Combined Ipilimumab and Nivolumab	PR	PR	Liver	1	Yes	Yes	Yes
P13	0	WT	Combined Ipilimumab and Nivolumab	PR	PR	Adrenal and liver	1	Yes	Yes	Yes
P14	0	V600 Mutant	Combined Ipilimumab and Nivolumab	SD	PR	Peritoneal and mesenteric nodes	3	Yes	Yes	Yes
P15	1	WT	Combined Ipilimumab and Nivolumab	PD	* PD	Inguinal and iliac nodes	2	Yes	Yes	Study halted due to COVID-19 crisis from March 2020

ECOG = Eastern Cooperative Oncology Group. Treatment response was evaluated based on the Response Evaluation Criteria in Solid Tumors (RECIST) guidelines version 1.1 i.e. Complete Response (CR); Partial Response (PR); Stable Disease (SD); Progressive Disease (PD)
** Represents trial patients who died following disease progression within the first 1 year of follow-up.*

Table S2. MRI sequence parameters.

**Parameter**	**T_2W**	**DKI**	**DCE**
Sequence	SSFSE	2D DW EPI	3D FSPGR
TR (ms)	1073-3231	4000-6667	3.2-3.3
TE (ms)	87.6-90.6	92.3-95.3	1.2-1.3
Flip angle (º)	90	90	16
Slice thickness (mm)	6	6	5
Slice gap (mm)	0	0	0
FOV (cm)	28-36	28-36	30-35
Image matrix	256 x 256	128 x 128	160 x 160 x N
Fractional k-space coverage	0.54	1	0.52-0.73
Parallel imaging factor	-	2	2.5
Acquisition time (min)	3:39	11:30	8:07

T_2W = T_2-weighted anatomical scans; DKI = diffusion kurtosis imaging; DCE = dynamic contrast-enhanced MRI; SSFSE = single-shot fast spin-echo; 2D DW EPI = two-dimensional diffusion-weighted echo planar imaging; 3D FSPGR = three-dimensional fast spoiled gradient-recalled echo; TR = repetition time; TE = echo time; FOV = field of view

Figure S1

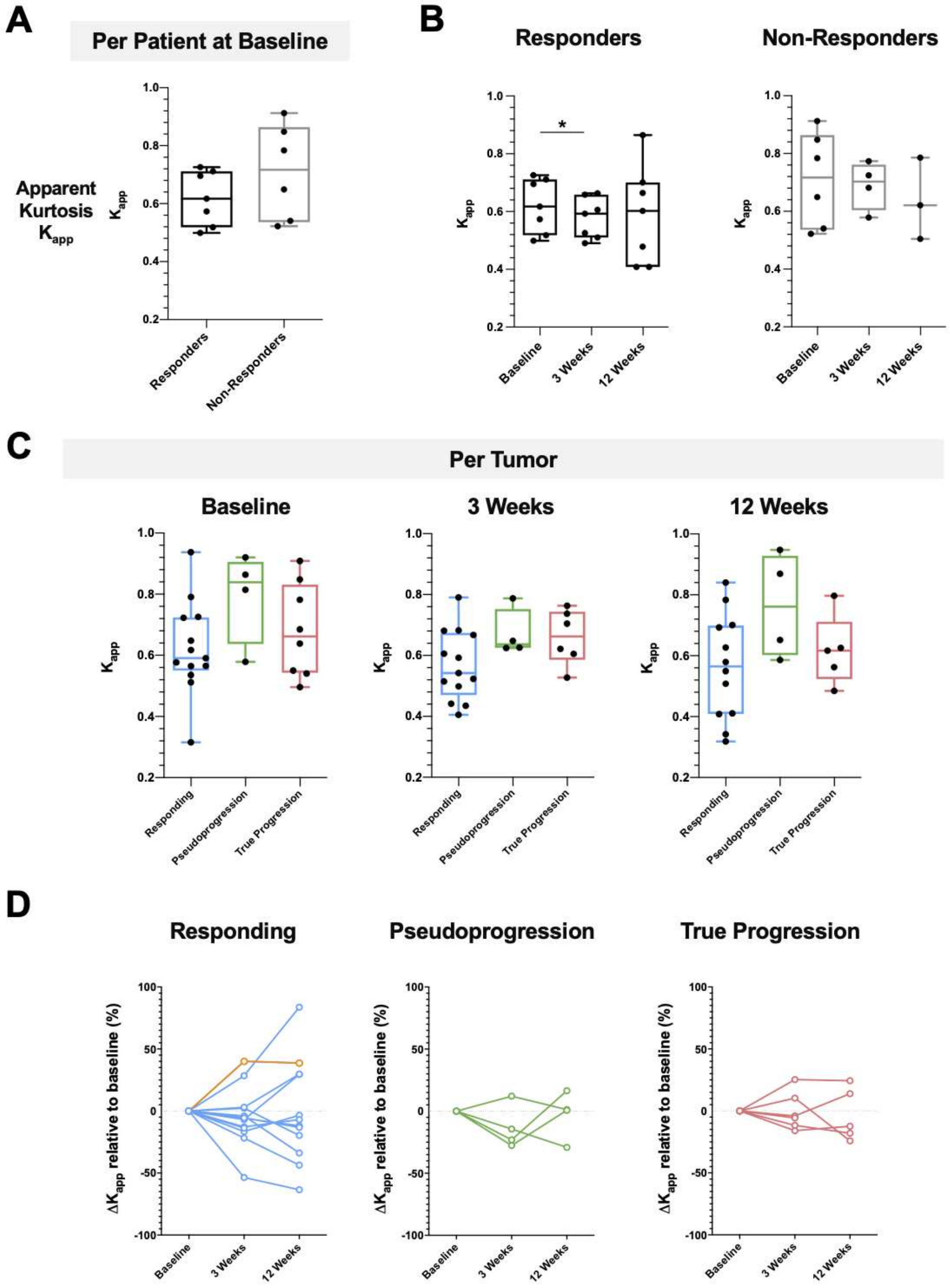
A
Per Patient at Baseline
Apparent Kurtosis Kapp
Kapp
Responders
Non-Responders
B
Responders
Non-Responders
Baseline
3 Weeks
12 Weeks
C
Per Tumor
Baseline
3 Weeks
12 Weeks
Responding
Pseudoprogression
True Progression
D
Responding
Pseudoprogression
True Progression
ΔKapp relative to baseline (%)

Figure S1

Measurement of tumor heterogeneity using DKI. (**A**) Comparison of apparent kurtosis (K_{app}) as a measure of tumor heterogeneity between responders and non-responders at baseline before the start of treatment. (**B**) Changes in tumor K_{app} among the patients over the course of treatment. (**C**) Differences in tumor heterogeneity among the three subgroups of individual lesions at baseline. (**D**) Percentage change in D_{app} relative to baseline in individual lesions from the three subgroups. * $p < 0.05$. Yellow line in (D) indicates the percentage change in K_{app} for patient P4.

Figure S2

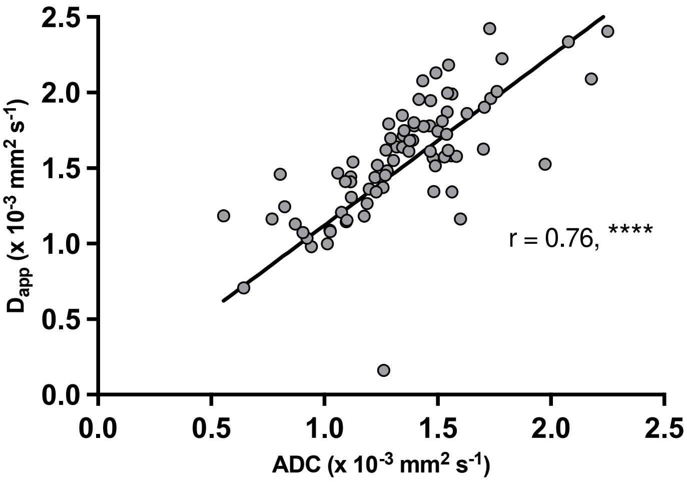

Spearman's correlation analysis showed a positive correlation between the apparent diffusivity value D_{app} measured on DKI and apparent diffusion coefficient ADC measured on DWI. **** $p < 0.0001$.

Figure S3

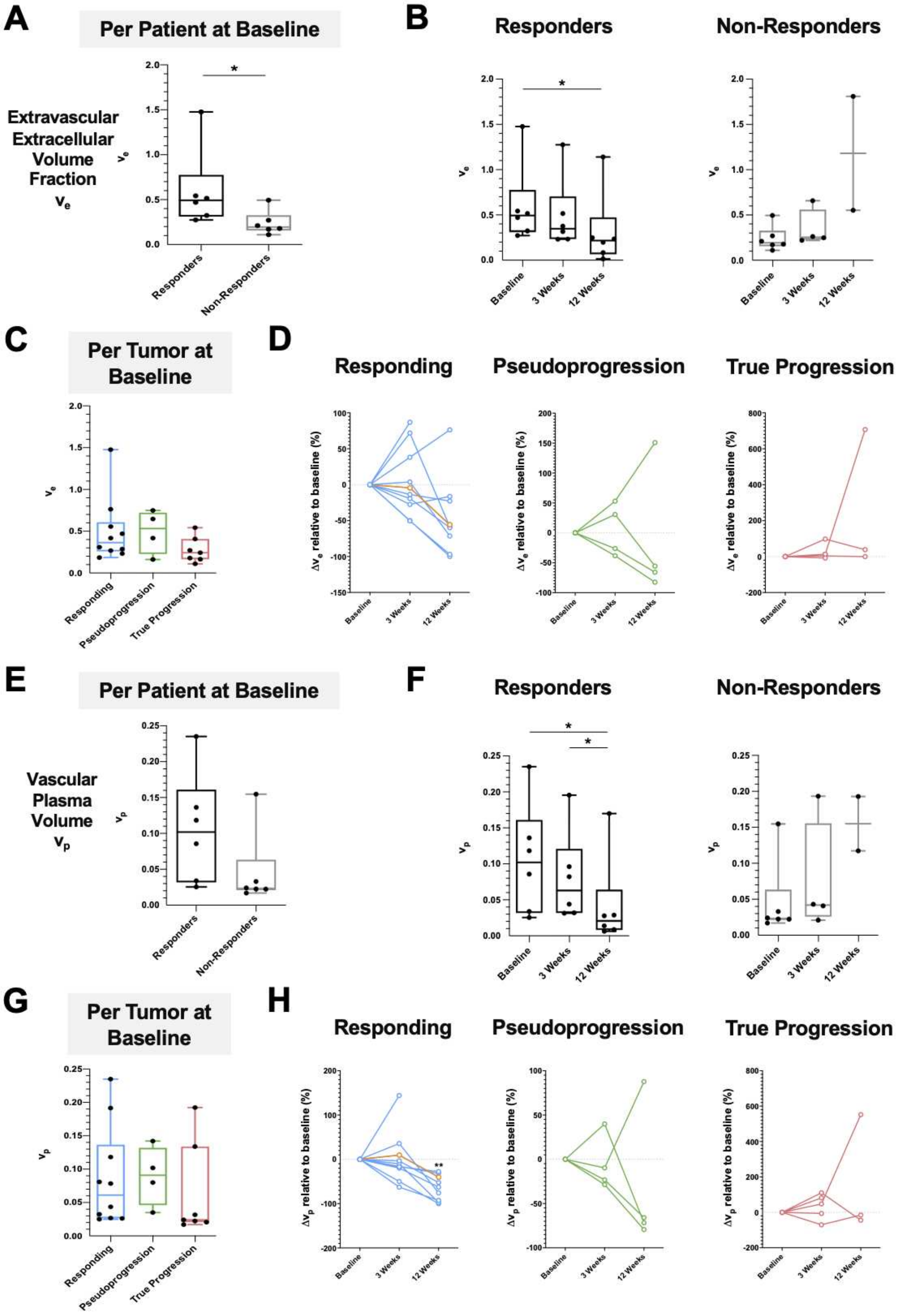

A
Per Patient at Baseline
Extravascular Extracellular Volume Fraction v_e
Responders
Non-Responders
B
Responders
Non-Responders
Baseline
3 Weeks
12 Weeks
C
Per Tumor at Baseline
Responding
Pseudoprogression
True Progression
D
Responding
Pseudoprogression
True Progression
Δv_e relative to baseline (%)
E
Per Patient at Baseline
Vascular Plasma Volume v_p
F
Responders
Non-Responders
G
Per Tumor at Baseline
H
Responding
Pseudoprogression
True Progression
Δv_p relative to baseline (%)

Comparison of changes in DCE-MRI parameters following treatment with immune checkpoint inhibitors among patients and within individual lesions: v_e, extravascular extracellular volume fraction (A-D); and v_p, vascular plasma volume (E-H). * $p < 0.05$; ** $p < 0.01$. Yellow line in (D) and (H) indicates the percentage change in v_e and v_p for patient P4.

Figure S4

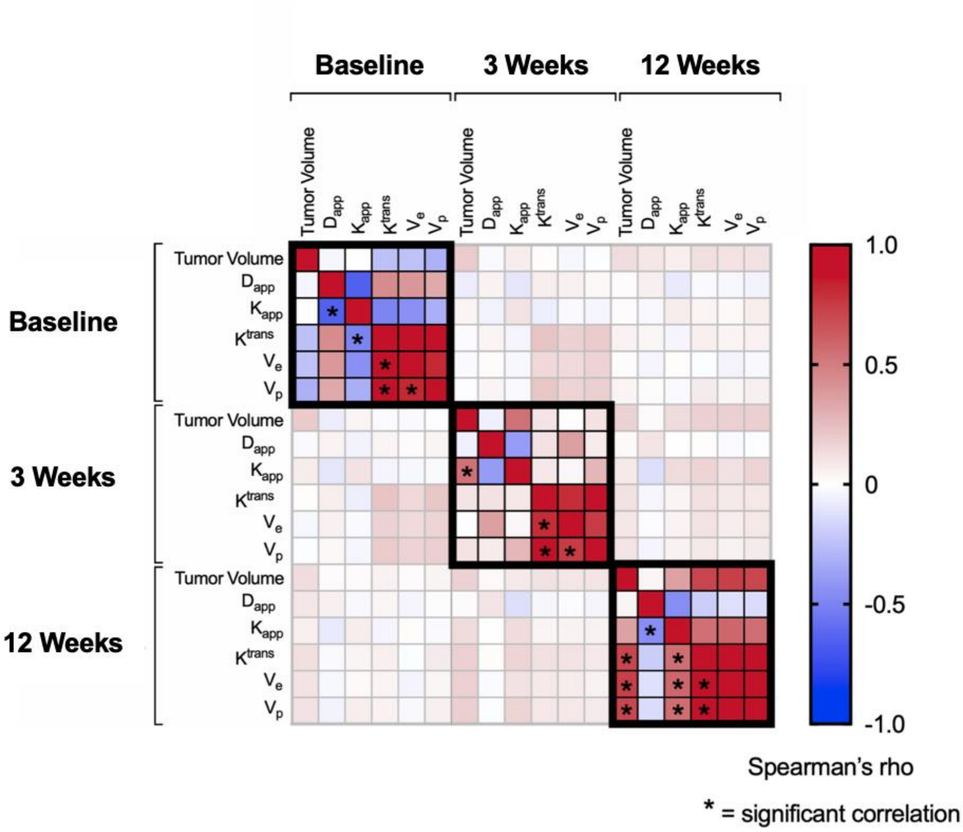

Spearman's correlation analysis of the MRI imaging biomarkers during the first 12 weeks of immune checkpoint blockade.

Supplemental Methods

MRI Image Acquisition

T_2-weighted SSFSE images were acquired in coronal, axial, and sagittal orientations (TE/TR = 90.6/1508 ms; slice thickness/gap 6.0/0.0 mm; matrix 256 x 256, NEX 0.54). Axial DKI was acquired using dual-echo EPI (TE/TR = 92.3/6000 ms; slice thickness/gap 6.0/0.0 mm; matrix 128 x 128) with five different *b*-values (100, 500, 900, 1300 and 1700 s/mm^2). A *b*-value of 0 s/mm^2 was collected but not used as low *b*-values have been reported to be susceptible to pseudo-perfusion bias that can be avoided using *b*-value >100 s/mm^2. Standard diffusion-weighted imaging (DWI) was performed using *b*-values of 100, 500, 900 s/mm^2. DCE-MRI was performed using a three-dimensional segmented k-space spoiled gradient-echo (3D SPGR) technique in a sagittal orientation. Dynamic phases were acquired at a temporal resolution of 12 s, which included approximately 7 s of scanning and a 5 s gap. The subjects were trained to hold their breath during the gradient noise and to breathe in and out during the periods of silence (TR/TE = 1.2/3.2 ms; flip angle 16°; slice thickness/gap 5.0/0.0 mm; matrix 160 x 160 x number of slices). A bolus injection of the intravascular contrast agent Gadobutrol (Gadovist, Bayer Pharma AG, Berlin, Germany) was administered at 0.1 mmol/kg intravenously 25 s after the start of acquisition at a rate of 3 ml/s, followed by 25 ml of saline flush using an MR-compatible power injector. Pre-contrast T_1-weighted gradient-echo images were acquired with five different flip angles (2°, 3°, 5°, 10°, 15°) and Bloch-Siegert B_1 maps[1] were obtained for subsequent B_1 inhomogeneity correction and T_1 mapping for analysis of the dynamic sequences.

Image Processing and Analysis

Quantitative maps of D_{app} were calculated from the DKI images using an in-house Matlab Script (Mathworks, MA, USA). Non-Gaussian water movements were quantified using a dimensionless metric termed kurtosis, governed by the polynomial equation (**Equation 1**):

$$S_i = S_0 \cdot \exp(-b_i \cdot D_{app}) \cdot \exp\left(\frac{1}{6} \cdot {b_i}^2 \cdot {D_{app}}^2 \cdot K_{app}\right) \tag{1}$$

where s_i is the signal intensity at b-value b_i, s_0 is the estimated signal intensity at *b*-value b_i = 0 s/mm^2 when no diffusion gradient is applied, D_{app} is the apparent diffusivity (s/mm^2) at *b*-values more than 1000 s/mm^2, and K_{app} is the apparent diffusion kurtosis (unitless)[2,3].

Quantitative maps of tumor vascular permeability and perfusion were calculated from the DCE images using MISTAR software (Apollo Medical Imaging Technology, Melbourne, Australia). B_1 maps of field inhomogeneity were calculated using in-house Matlab scripts for subsequent B_1-correction of pre-contrast T_1 maps. A model arterial input function (AIF) based on Fritz-Hansen *et al.*[4] and the extended Tofts model [5,6] were used for pharmacokinetic modelling of the intravascular contrast enhancement within tissues. The Fritz-Hansen model AIF was used with values after the first pass extrapolated from the Weinmann function [7], scaled accordingly. Total tissue contrast concentration as a function of time $[C_e(t)]$ can be approximated using (**Equation 2**):

$$C_e(t) = \nu_p C_p(t) + K^{trans} \int_0^t C_p(t)\, e^{-k_{ep}(t-\tau)}\, d\tau \tag{2}$$

where $C_p(t)$ is the concentration in blood plasma as a function of time as determined by the AIF measured in a large feeding vessel, K^{trans} is the volume transfer coefficient from the blood plasma space into the extravascular tumor interstitial space reflecting vascular permeability, v_p is the vascular plasma volume, v_e is the fractional volume of the extravascular-extracellular space, and k_{ep} is the flux rate constant These parameters are interrelated as follows:

$$K^{trans} = v_e \times k_{ep} \quad (3)$$

Motion correction was performed on all images using in-house Matlab scripts prior to the calculation of quantitative maps.

Volumetric tumor regions of interest (VOIs) were drawn on the T_2-weighted images and quantitative maps of D_{app}, K_{app}, K^{trans} v_e and v_p by a radiologist (FS) with 3 years of experience in the oncological setting and blinded to the clinical outcome and a radiology researcher (DL) with 4 years of experience in oncological imaging. VOIs from each tumor were expressed as single T_2 volume, D_{app}, K_{app}, K^{trans} v_e and v_p values and volume-weighted for analysis for the patient as a whole or for individual lesion. T_2-weighted images taken at each imaging timepoints were normalized based on intensity scaling to the baseline images before calculating the T_2 histograms[8].

Immunohistochemistry

All immunochemistry (IHC) procedures were performed on histologically confirmed melanoma tissues obtained for diagnosis. Tissues were fixed with 10% buffered formalin solution and processed into paraffin embedded (FFPE) tissue blocks. Serial tissue sections of

3.5 µm thickness were prepared from the FFPE blocks and mounted onto silanized slides at Addenbrooke's Hospital Human Research Tissue Bank. IHC and semi-automated image analysis were carried out at AstraZeneca UK. Slides were baked for 1 h at 60ºC, deparaffinized in xylene and rehydrated with decreasing concentrations of ethanol. Automated H&E staining was performed on Leica ST5020 Multistainer (Leica Biosystems, Germany). Immunostaining for CD31, Ki67, CAIX, CD8, FOXP3 and CD11b was conducted on a Ventana Benchmark ULTRA automated slide processing system (Ventana Medical Systems Inc., Roche Tissue Diagnostics, Germany) according to the manufacturer's instructions. Briefly, heat-induced epitope retrieval in Tris-EDTA buffer pH 7.8 at 95ºC for 44 min was carried out on all slides using Ventana's ULTRA Cell Conditioning 1 (CC1) solution, and endogenous peroxidase activity was quenched using DISCOVERY Inhibitor (Roche Tissue Diagnostics, Germany) at 37ºC for 4 min. Primary antibodies incubation was conducted at 37ºC for 1 h using the following monoclonal anti-human antibodies: mouse anti-human CD31 (1 µg/ml, clone JC70A, Dako, Denmark), CONFIRM™ rabbit anti-human Ki67 (2 µg/ml, clone 30-9, Roche Tissue Diagnostics, Germany), rabbit anti-human carbonic anhydrase IX (CAIX) (2 µg/ml, clone EP161, Roche Tissue Diagnostics, Germany), rabbit anti-human CD8 (0.58 µg/ml, clone SP239, Spring Bioscience, USA), rabbit anti-human Foxp3 (0.20 µg/ml, clone SP97, Spring Bioscience, USA) and rabbit anti-human CD11b (0.03 µg/ml, clone D6X1N, Cell Signaling Technology, USA). This was followed by secondary antibodies incubation at 37ºC for 16 min using either DISCOVERY™ high quality horseradish peroxidase (HQ-HRP) kit for highest sensitivity and specificity in detection and chromogen staining using EnVision FLEX HRP Magenta Substrate Chromogen System (Dako, Denmark), DISCOVERY™ Purple HRP-activated Chromogen kit or DISCOVERY™ Teal HRP-activated Chromogen kits (Roche Tissue Diagnostics, Germany). Human tonsil, spleen and placenta were used as positive control tissues. Immunostaining with mouse or

rabbit negative isotype control antibodies (Roche Tissue Diagnostics, Germany) was used as negative controls. Slides were then counterstained with hematoxylin. All stained slides were scanned at 20X and 40X magnification on Aperio AT2 (Leica Biosystems, Germany). Semi-automated image analysis of the whole tissue slides was performed on HALO® software (Indica Labs, USA).

References

1. Sacolick LI, Wiesinger F, Hancu I, Vogel MW. B 1 mapping by Bloch-Siegert shift. Magn Reson Med. 2010;63(5):1315–22.
2. Deen SS, Priest AN, McLean MA, Gill AB, Brodie C, Crawford R, et al. Diffusion kurtosis MRI as a predictive biomarker of response to neoadjuvant chemotherapy in high grade serous ovarian cancer. Sci Rep. 2019;9(1):1–9.
3. Goodburn RJ, Barrett T, Patterson I, Gallagher FA, Lawrence EM, Gnanapragasam VJ, et al. Removing rician bias in diffusional kurtosis of the prostate using real-data reconstruction. Magn Reson Med. 2020 Jun 18;83(6):2243–52.
4. Fritz-Hansen T, Rostrup E, Larsson HBW, Søndergaard L, Ring P, Henriksen O. Measurement of the arterial concentration of Gd-DTPA using MRI: A step toward quantitative perfusion imaging. Magn Reson Med. 1996;36(2):225–31.
5. Tofts PS. Modeling tracer kinetics in dynamic Gd-DTPA MR imaging. J Magn Reson Imaging. 1997;7(1):91–101.
6. Paul ST, Gunnar B, David LB, Jeffrey LE, Elizabeth H, Michael VK, et al. Estimating kinetic parameters from dynamic contrast-enhanced t1-weighted MRI of a diffusable tracer: Standardized quantities and symbols. J Magn Reson Imaging. 1999;10(3):223–32.
7. Weinmann HJ, Laniado M, Mützel W. Pharmacokinetics of GdDTPA/dimeglumine after intravenous injection into healthy volunteers. Physiol Chem Phys Med NMR. 1984;16(2):167–72.
8. Sun X, Shi L, Luo Y, Yang W, Li H, Liang P, et al. Histogram-based normalization technique on human brain magnetic resonance images from different acquisitions. Biomed Eng Online. 2015 Dec 28;14(1):73.
